# The Predictive Value of Donor Renal Functions, Graft Weight, Recipient Body Mass Index, and Human Leucocyte Antigen Match Regarding Early Graft Functions

**DOI:** 10.7759/cureus.35438

**Published:** 2023-02-25

**Authors:** Onur Açıkgöz, Serkan Akıncı

**Affiliations:** 1 Urology, Memorial Hizmet Hospital, Istanbul, TUR

**Keywords:** chronic kidney failure, kidney disease, kidney transplant recipients, live donor, • kidney transplantation

## Abstract

Background and objective

Graft performance is the most important postoperative parameter for patients undergoing kidney transplantation (KTx). The renal function of the donor is reported to be correlated with graft function after KTx. The body mass index (BMI) is also one of the important parameters involved in the prediction of graft function. The aim of this retrospective study was to examine the relationship between early postoperative graft function in patients undergoing KTx and donor cystatin C and estimated glomerular filtration rate (e-GFR) levels, graft weight/recipient BMI (G/B) ratio, and human leukocyte antigen (HLA) tissue compatibility.

Materials and methods

A total of 215 cases (215 donors, 215 recipients) who underwent KTx at our center between January 2018 and December 2022 were included in the study. Patients' age, sex, BMI, preoperative donor serum cystatin C and e-GFR levels, HLA tissue compatibility, graft weights, and recipient creatinine levels were recorded one week postoperatively. The Kolmogorov-Smirnov test and histogram plots were used to analyze the conformity of the variables to the normal distribution and Spearman's correlation test was used to analyze the relationship between variables.

Results

A negative correlation was identified between recipient creatinine level and G/B ratio and donor e-GFR (r = −0.256 and −0.137, respectively). Donor cystatin C level showed a positive correlation with recipient creatinine level (r = 0.242). No significant correlation was noted in terms of tissue matching rates (p = 0.616).

Conclusion

Although these three parameters are correlated with early graft functions, the graft weight/recipient BMI ratio has the strongest correlation.

## Introduction

Kidney transplantation (KTx) is an effective treatment strategy for patients with end-stage renal disease (ESRD) [1]. Graft performance is the most important postoperative parameter for patients undergoing KTx. Hence, graft function prediction is important for proper postoperative management based on the expected clinical course for the patient. Early postoperative graft function depends on numerous donor- and recipient-related functional, anatomical, and immunologic factors. Furthermore, postoperative immunosuppressive and conservative treatment regimens are known to have a significant effect on graft performance [2].

The renal function of the donor is reported to be correlated with graft function after transplantation [3]. The expectation that good preoperative renal function of the donor will positively affect graft function in the recipient can be considered reasonable. Additionally, in studies that evaluated the effect of the volume/size of the transplanted kidney on postoperative graft function, this parameter has been reported to be a strong preliminary indicator [4]. The body mass index (BMI) is also one of the important parameters involved in the prediction of graft function. Donor and recipient BMI affect postoperative graft function [5,6]. However, of these established and routinely used parameters, it is unclear which has a stronger predictive value for early recipient graft function.

The aim of this retrospective study was to examine the relationship between early postoperative graft function in patients undergoing KTx and donor cystatin C and estimated glomerular filtration rate (e-GFR) levels, graft weight/recipient BMI ratio, and human leukocyte antigen (HLA) tissue compatibility.

## Materials and methods

This study was conducted in accordance with the principles of the Declaration of Helsinki and was approved by the local ethics committee (Approval no: 2023-01-30).

The data of patients who underwent KTx at our center between January 2018 and December 2022 were retrospectively reviewed. After excluding patients whose archival records could not be accessed, patients who developed acute rejection in the early period, and patients receiving cadaveric transplants, a total of 215 KTx cases (215 donors, 215 recipients) were included in the study. Cadaveric kidney transplants were excluded because all pertinent parameters were not evaluated in those cases. Furthermore, the delayed graft function rate - observed at a higher probability in cadaveric transplants compared to living transplants - could have caused interpretation errors in the statistical analyses.

Patients' age, sex, BMI, preoperative donor serum cystatin C and e-GFR levels, HLA tissue compatibility, graft weights, and recipient creatinine levels were recorded one week postoperatively. Graft weights were acquired from data recorded by weighing the graft kidney after removing perinephric adipose tissue following bench surgery. The Chronic Kidney Disease Epidemiology Collaboration (CKD-EPI) formula was used to calculate e-GFR. The effect of graft weight on postoperative graft function was evaluated by calculating the graft weight (g)/recipient BMI (kg/m^2^) (G/B) ratio. HLA tissue compatibility was statistically analyzed by examining the number of matches in the three HLA pairs (A, B, DR).

All patients were operated on by the same surgical team using the same surgical technique. In the postoperative period, all patients received the routine immunosuppressive treatment regimen administered at our center. Induction and maintenance of the immunosuppression treatment were performed in all patients; this treatment comprised antithymocyte globulin (ATG, 3 mg/kg, three days), tacrolimus (FK506, 1 mg/kg), mycophenolate mofetil (2 × 1000 mg), and prednisolone. The doses were adjusted for a target blood level of 10 mg/ml for FK506 and a target CD3 count of 20-50 for ATG.

Statistical analyses were performed using the IBM SPSS Statistics version 25.0 (IBM Corp., Armonk, NY). The Kolmogorov-Smirnov test and histogram plots were used to analyze the conformity of the variables to normal distribution. Mean, standard deviation (SD), and median (min-max) values were used when presenting descriptive analyses. Spearman's correlation test was used to analyze the relationship between variables. P-values below 0.05 indicate statistical significance.

## Results

Table 1 summarizes patient demographic data and descriptive statistics. The mean recipient age was 42.8 years and the mean donor age was 46.7 years; the female/male ratio was 82/133 in recipients and 117/98 in donors. The mean G/B ratio of the patients included in the study was 7.06; the mean donor cystatin C and e-GFR levels were 0.8 mg/L and 106.4 ml/min, respectively; the mean HLA tissue compatibility was 2.4; and the mean creatinine level of recipients at postoperative week one was 1.4 mg/dL (Table 1).

**Table 1:** Overall characteristics of patients SD: standard deviation; G/B ratio: graft weight/recipient body mass index ratio; e-GFR: estimated glomerular filtration rate; HLA: human leucocyte antigen

Variables	Values
Recipient age, years	
Mean ±SD	42.8 ±13.4
Median (range)	43 (11-71)
Recipient sex, n (%)	
Male	133 (61.8)
Female	82 (38.1)
Donor age, years	
Mean ±SD	46.7 ±12.5
Median (range)	46 (22-81)
Donor sex, n (%)	
Male	98 (45.5)
Female	117 (54.4)
G/B ratio	
Mean ±SD	7.0 ±2.1
Median (range)	6.6 (3.2-18.4)
Donor cystatin C, mg/L	
Mean ±SD	0.8 ±0.1
Median (range)	0.8 (0.4-1.9)
Donor e-GFR, mL/min	
Mean ±SD	106.4 ±13.3
Median (range)	106.1 (64.2-137.3)
HLA compatibility	
Mean ±SD	2.4 ±1.6
Median (range)	3 (0-6)
Recipient creatinine, week 1, mg/dL	
Mean ±SD	1.4 ±0.6
Median (range)	1.3 (0.4-6.1)

**Figure 1:**
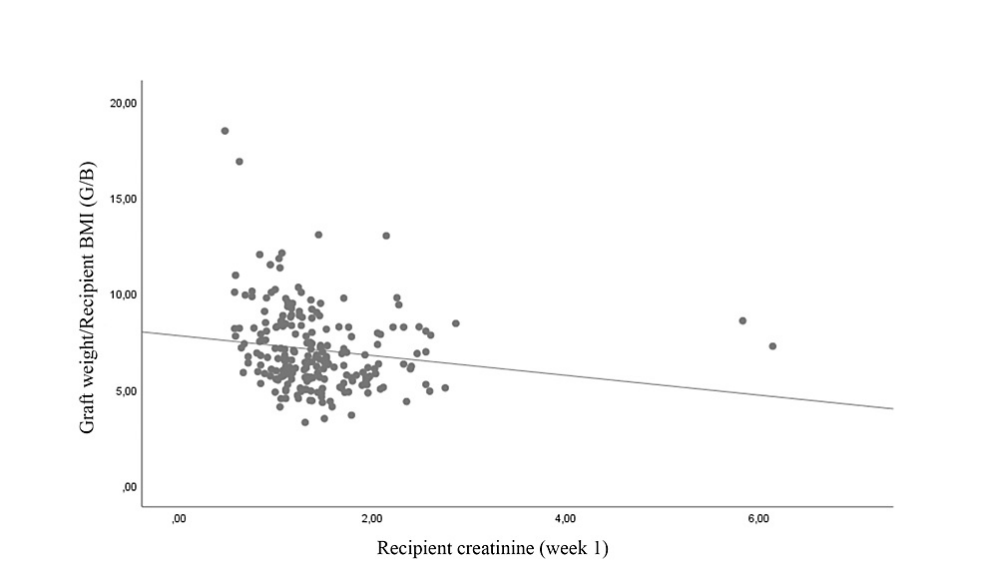
Correlation of graft weight/recipient BMI ratio with recipient creatinine BMI: body mass index

**Figure 2:**
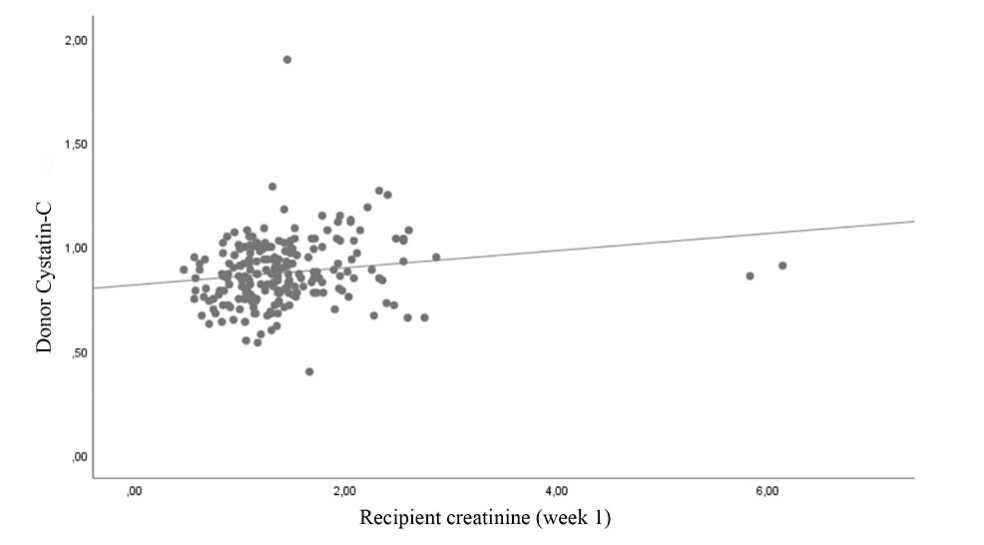
Correlation of donor cystatin C level with recipient creatinine

**Figure 3:**
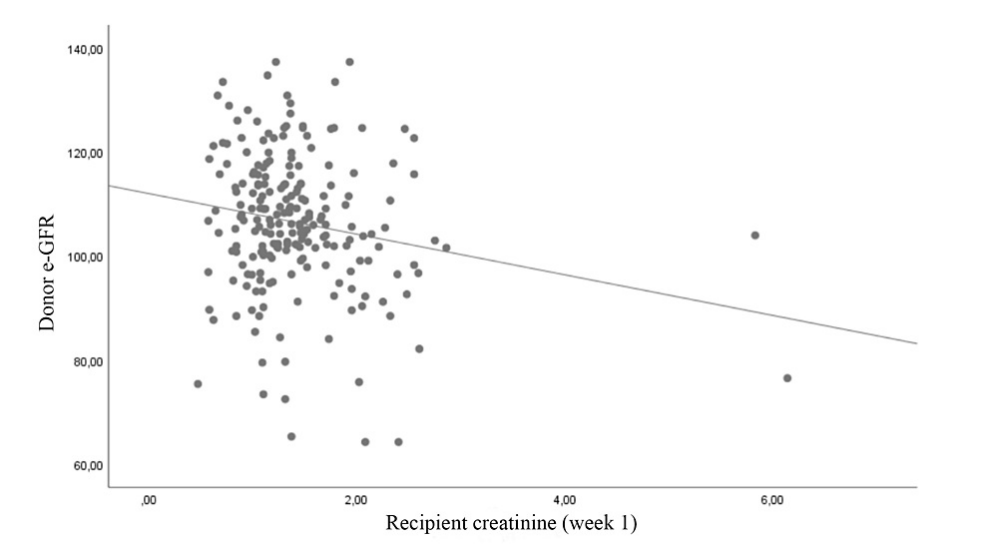
Correlation of donor e-GFR level with recipient creatinine e-GFR: estimated glomerular filtration rate

**Figure 4:**
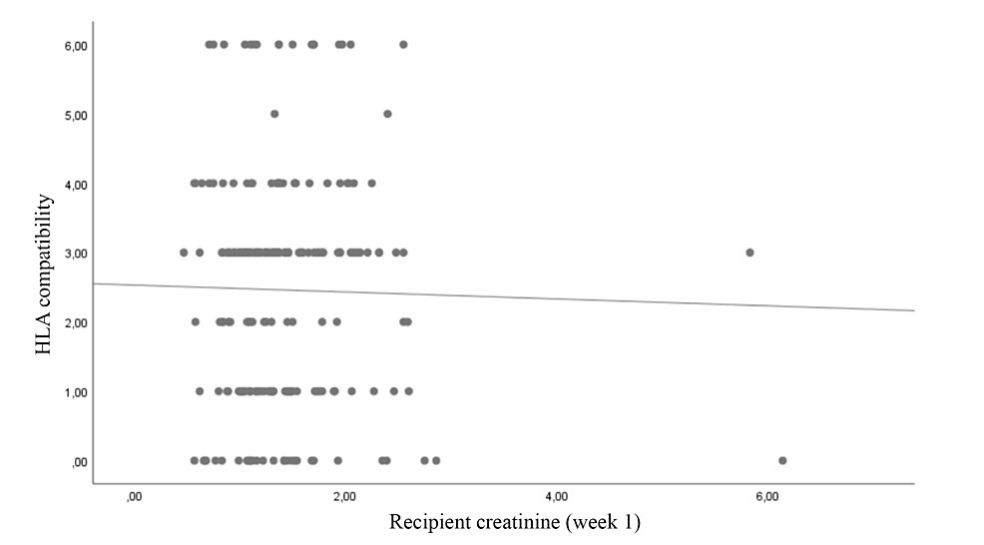
Correlation of HLA match with recipient creatinine HLA: human leucocyte antigen

The correlation between recipient creatinine level and donor cystatin C level, donor e-GFR level, G/B ratio, and HLA compatibility at postoperative week one was examined. A negative correlation was identified between recipient creatinine level and G/B ratio and donor e-GFR (r = −0.256 and −0.137, respectively). Donor cystatin C level showed a positive correlation with recipient creatinine level (r = 0.242). No significant correlation was noted in terms of tissue matching rates (p = 0.616) (Table 2; Figures 1, 2, 3, 4).

**Table 2:** Results of correlation analysis *Spearmen’s correlation analysis G/B ratio: graft weight/recipient body mass index ratio; e-GFR: estimated glomerular filtration rate; HLA: human leucocyte antigen

Variables	Recipient creatinine	
	r^*^	p^*^
G/B ratio	-0.256	<0.001
Donor cystatin C	0.242	<0.001
Donor e-GFR	-0.137	0.045
HLA compatibility	0.034	0.616

## Discussion

Although numerous parameters can be used to predict graft function before KTx, the most important parameter is the preoperative renal function of the donor [7]. In the preoperative period, e-GFR assays are often used to evaluate donor kidney function. Hawley et al. investigated the effect of donor e-GFR on recipient graft functions and emphasized that it is the most important parameter in predicting graft function [3]. The authors stated that they used the Cockcroft-Gault formula to measure the e-GFR. There are several other formulas to estimate renal function. Although a limited number of studies have reported that some formulas are superior in certain aspects, they generally measure GFR with a similar degree of success [8-10]. In addition, Sberro et al. conducted a comparison of e-GFR measurement based on the Cockcroft-Gault formula and concluded that graft functions can be predicted preoperatively [11]. In the present study, e-GFR was measured using the CKD-EPI method and a significant correlation was noted between e-GFR graft function in the early period.

Cystatin C is a low-molecular-weight proteinase inhibitor and is considered an endogenous GFR marker. Furthermore, its cellular production rate is constant, it undergoes free filtration in the glomeruli, its serum level is not affected by other diseases and, as a test, it can rapidly yield results [12]. Many studies have reported that it can be used in KTx both in the evaluation of postoperative graft functions and in the preoperative GFR evaluation in donor candidates [13-16]. However, the effect of donor cystatin C level on early graft function may require further studies. Considering the fact that cystatin C levels provide reliable information associated with renal function in general, graft function can be expected to be good when the donor has low cystatin C levels. In the present study, the relationship between donor cystatin C level and recipient post-KTx week-one creatinine value was consistent with this expectation.

In our study, a comparison of the effects of two renal function markers (e-GFR and cystatin C) on early recipient serum creatinine level revealed that the relationship between cystatin C level and graft function was relatively stronger (r = 0.242 vs. −0.137) than that between e-GFR and graft function. Studies evaluating cystatin C have reported that it is not affected by muscle mass, sex, and body surface area [17,18]. The stronger correlation between early graft function and cystatin C compared to e-GFR may be explained by the fact that serum cystatin C level is a more sensitive marker of renal function [19,20].

Various studies have reported that recipient BMI has a significant effect on graft function, especially in the early period [21-23]. Xin et al. retrospectively evaluated 2462 KTx cases and reported that recipient BMI was an independent factor in predicting graft function [22]. Similarly, Zhao et al. noted that donor GFR and recipient body weight were independent factors affecting renal function in the second year after transplantation [23]. Some studies in the literature have evaluated the BMI or body weights of the donor and recipient together, rather than focusing only on the recipient's BMI. Špatenka et al. showed that the donor/recipient body weight ratio is correlated with graft function in the pediatric population [24]. Additionally, Zheng et al. calculated the ratio of the donor and recipient body surface area (D/R BSA) and showed that post-transplant graft functions were significantly better in kidney transplants with a D/R BSA ratio of >1.2 [25]. Studies including the weight or size of the grafted kidney in addition to BMI and similar parameters (body weight and body surface area-BSA) of the recipient have reported a strong correlation with graft function. Al-Adra et al. evaluated 438 living KTx cases and reported that renal volume was an independent predictor of recipient e-GFR [4]. Lee et al. examined the ratio of kidney volume to recipient BSA and reported that kidney volume/BSA ratio together with donor age were independent indicators for predicting graft function [26]. A similar study was conducted by Song et al. on 213 patients. In this study, however, the ratio of kidney weight to recipient body weight (Kw/Rw) was evaluated instead of kidney volume. The authors reported that the Kw/Rw ratio was correlated with graft function up to five years after kidney transplantation [27]. In the present study, graft weight was examined as an indirect indicator of the amount of functional nephrons, and the graft weight/recipient BMI (G/B) ratio was evaluated to obtain a more effective parameter. The G/B ratio had a significant effect on early postoperative graft function. Correlation analysis showed that the G/B ratio had a relatively stronger correlation with the recipient post-KTx week-one creatinine level compared to cystatin C and e-GFR (r= -0.256 vs. 0.242 and -0.137).

HLA tissue compatibility, which was evaluated for the prediction of early graft function, did not show a statistically significant correlation with the recipient’s creatinine level at one week post-KTx (p = 0.616). It is known that HLA tissue compatibility is an important factor affecting long-term graft function [28]. High-dose immunosuppressive therapy administered to patients who have undergone KTx, especially in the early postoperative follow-up, seems to have overshadowed the effect of HLA tissue compatibility on graft function within this period.

Among the parameters examined in the present study, the G/B ratio showed the strongest correlation with early graft function, followed by donor cystatin C level and e-GFR, respectively. The main limitations of the study are its single-center and retrospective design and the exclusion of some immunological factors, comorbidities, and certain parameters (e.g., duration of dialysis) that may have had an impact on graft function in the statistical analysis. The main reason for this exclusion was to compare the predictive ability of the parameters we focused on, rather than determining the parameters that affect the graft functions.

## Conclusions

Donor serum cystatin C and e-GFR levels are strongly correlated with early postoperative serum creatinine levels in patients who have undergone kidney transplantation. Graft weight and recipient BMI are also parameters that can be used for the estimation of graft functions, and it is possible to use the “graft weight/recipient BMI” ratio in order to use both parameters effectively. Although these three parameters are correlated with early graft functions, the graft weight/recipient BMI ratio has the strongest correlation. Multicenter studies with a prospective design are needed to validate the findings obtained in the present study.
